# Inducible Arginase 1 Deficiency in Mice Leads to Hyperargininemia and Altered Amino Acid Metabolism

**DOI:** 10.1371/journal.pone.0080001

**Published:** 2013-11-04

**Authors:** Yuan Yan Sin, Laurel L. Ballantyne, Kamalika Mukherjee, Tim St. Amand, Lianna Kyriakopoulou, Andreas Schulze, Colin D. Funk

**Affiliations:** 1 Department of Biomedical and Molecular Sciences, Queen's University, Kingston, Ontario, Canada; 2 Division of Clinical and Metabolic Genetics, and Research Institute, The Hospital for Sick Children, University of Toronto, Toronto, Ontario, Canada; INRA, France

## Abstract

Arginase deficiency is a rare autosomal recessive disorder resulting from a loss of the liver arginase isoform, arginase 1 (ARG1), which is the final step in the urea cycle for detoxifying ammonia. ARG1 deficiency leads to hyperargininemia, characterized by progressive neurological impairment, persistent growth retardation and infrequent episodes of hyperammonemia. Using the Cre/loxP-directed conditional gene knockout system, we generated an inducible Arg1-deficient mouse model by crossing “floxed” Arg1 mice with CreER^T2^ mice. The resulting mice (Arg-Cre) die about two weeks after tamoxifen administration regardless of the starting age of inducing the knockout. These treated mice were nearly devoid of Arg1 mRNA, protein and liver arginase activity, and exhibited symptoms of hyperammonemia. Plasma amino acid analysis revealed pronounced hyperargininemia and significant alterations in amino acid and guanidino compound metabolism, including increased citrulline and guanidinoacetic acid. Despite no alteration in ornithine levels, concentrations of other amino acids such as proline and the branched-chain amino acids were reduced. In summary, we have generated and characterized an inducible Arg1-deficient mouse model exhibiting several pathologic manifestations of hyperargininemia. This model should prove useful for exploring potential treatment options of ARG1 deficiency.

## Introduction

The urea cycle disorders (UCDs) are a group of rare inborn errors of hepatic metabolism causing malfunction in the nitrogen clearance system. Deficiency in any of the enzymes in the urea cycle results in perturbation of ureagenesis, thereby leading to incomplete removal of ammonia from the blood stream and to variable degrees of hyperammonemia. Arginase is the final enzyme of the urea cycle and is of central importance in the detoxification of ammonia in mammals. There are two isoforms of arginase which are present at distinct intracellular sites: arginase 1 (ARG1) and arginase 2 (ARG2). ARG1, which is expressed predominantly in the cytoplasm of the liver, hydrolyzes arginine into ornithine and urea, where the latter is then excreted through the kidneys in the urine [1], [2]. In contrast, the second isoform, mitochondrial ARG2 is distributed in extrahepatic tissues, such as kidney and prostate, with lower levels in brain, gastrointestinal tract and macrophages [2], [3]. In patients with ARG1 deficiency and hyperammonemic episodes, there is often a compensatory increase in ARG2 activity in the kidney [4]. Although the exact mechanism is not known, the augmented expression of ARG2 may mitigate the phenotypic deterioration via residual ureagenesis [1], [4].

In humans, arginase deficiency generally refers to decreased function of the liver arginase isoform, ARG1. This deficiency is transmitted in an autosomal recessive manner and often leads to hyperargininemia, a metabolic disorder characterized by progressive neurological and intellectual impairment, spasticity and persistent growth retardation [1]. Some patients may also display intermittent episodes of behavior disturbance (irritability, hyperactivity and aggression), feeding difficulties, vomiting, lethargy and seizures [5]. Unlike other urea cycle disorders with early-onset presentation, hyperargininemia usually presents after the neonatal period, particularly between two to four years of age with predominantly neurological manifestations and infrequent episodes of hyperammonemia. As ARG1-deficient patients rarely develop hyperammonemia crises, they have a prolonged life span compared to those with other urea cycle disorders. With early intervention, some of the neurologic abnormalities can be partially alleviated by low-protein diet, supplementation of essential amino acids and administration of nitrogen-scavenging drugs, such as sodium benzoate or sodium phenylbutyrate [1], [6]. However, no complete cure is currently available.

The ∼12 kb gene for human liver arginase (*ARG1*) is located on chromosome 6q23, composed of eight exons and seven introns [7], [8]. The genomic structure of *ARG1* reveals a high level of conservation with rodent *Arg1* [8]–[10]. Mice with homozygous disruption of *Arg1* were generated by Iyer and co-workers to study the pathological mechanisms of the disease [11]. Their gene knockout mouse model partially mimicked human conditions, characterized by severe hyperammonemia and neurological deterioration. However, unlike human subjects, which usually survive into adulthood, these knockout mice died in the perinatal period (between postnatal day 10–14). El Kasmi *et al*. [12] developed macrophage-specific Arg1 knockout mice to circumvent the neonatal mortality in order to study macrophage immune functions of Arg1.

Here, using the Cre/loxP-directed conditional gene knockout system [13], we report a new Arg1-deficient mouse model that allows inducible temporal control of *Arg1* deletion in mice. Through tamoxifen-induced excision of floxed exons 7 and 8 of *Arg1* at four different age groups (neonatal, 4-, 8- and 12-weeks), the resulting mice are shown to be nearly devoid of Arg1 activity. Such deletion exhibited several pathobiochemical aspects of hyperargininemia commonly seen in humans. We anticipate that our model will yield novel insights into the roles of Arg1 and address the development of potential gene therapeutic strategies for the treatment of this disorder.

## Materials and Methods

### Generation of knockout mice

*Arg1^flox^* (JAX strain 008817, C57BL/6-*Arg1^tm1Pmu^*/J) [12] and CreER^T2^ (JAX strain 008463, B6.129-*Gt(ROSA)26Sor^tm1(cre/ERT2)Tyj^*/J) [14] mice were obtained from The Jackson Laboratory and crossbred over two generations to obtain *Arg1^flox^* homozygous and CreER^T2^ homozygous or heterozygous offspring. Newborns at postnatal day 0 (P0) were intragastrically injected with 4-hydroxy-tamoxifen (OHT) (Sigma) for two consecutive days, while mice at 4-, 8-, and 12- week of age received five consecutive intraperitoneal injections of 1 mg tamoxifen (Sigma) [13] to induce excision of exons 7 and 8 of the *Arg1* gene. Control mice for side-by-side comparison include *Arg1^flox^* mice which received equal volumes of vehicle (5% ethanol in sunflower oil) (herein referred to as vehicle-treated mice) and Cre mice which received equal volumes of tamoxifen administration (herein referred to as ROSA mice). Changes in body weight and dietary intake of mice were carefully monitored daily during the experimental period. Food intake was measured by pre-weighing food pellets and then constantly weighing the remaining chow at the indicated times. All procedures were reviewed and approved by the Queen's University Animal Care Committee (approval #Funk-2011-048-R1-A4) and conformed to the Guidelines of the Canadian Council on Animal Care. Unless otherwise specified, water and standard rodent chow (protein 18.9%, fat 11%, fiber 2.2%, minerals 5.8%) (5015 Mouse Diet, Lab Diet)) were provided *ad libitum*. In early experiments, mice were treated with 1 ml of warmed Lactated Ringer's Solution (s.c.), which did not prove to be therapeutic. As the study progressed, it became possible to predict 24 h before mice would show signs of health deterioration and they were euthanized by CO_2_ asphyxiation before exhibiting signs of distress. Humane endpoints were defined as body weight loss of >15% relative to the weight at the time of tamoxifen administration, accompanied by hunched posture, lethargy and poor grooming. Some mice could lose substantial weight in one day especially when approaching the endpoint. For experimental consistency, all mice were sacrificed 14 days after tamoxifen administration. Approximately equal numbers of male and female mice were used for all studies. Harvested tissues were flash-frozen in liquid nitrogen and stored at −80°C prior to RNA isolation and arginase activity assay.

### PCR genotyping

Genomic DNA from ear punch or tail biopsy was extracted by standard protocols and subjected to PCR for genotyping. Primer sets for *Arg1^flox^* confirmation were as follows: 5′- TGCGAGTTCATGACTAAGGTT-3′ and reverse 5′-AAAGCTCAGGTGAATCGG-3′; for CreER^T2^: forward 5′-AAAGTCGCTCTGAGTTGTTAT-3′ and reverse 5′-GGAGCGGGAGAAATGGATATG-3′; and for Arg-Cre knockout: *Arg1^flox^* primer set in combination with reverse primer 5′-GCACTGTCTAAGCCCGAGAGTATC-3′. The removal of exons 7 and 8 was confirmed four days following tamoxifen administration. Cycle parameters: denaturation at 94°C for 30 sec, annealing at 64.5°C for 1 min, and elongation at 72°C for 1 min for 35 cycles.

### Gait analysis

Gait characteristics were analyzed eight days after tamoxifen administration by applying ink of different colors to the fore and hind paws, and letting them walk on a folded paper “alley”. The footprint patterns were analyzed for four parameters (all measured in centimeters): (i) stride length, as the average distance of forward movement between each stride; (ii) forepaw base width and (iii) hindpaw base width, as the average distance between left and right forepaws, and left and right hindpaws, respectively by measuring the perpendicular distance of a given footprint to a line connecting its opposite preceding and proceeding footprints; and (iv) distance from left or right forepaw/hindpaw overlap. Footprints made at the beginning and end of the run were excluded.

### Isolation of primary hepatocytes

Primary hepatocytes were isolated from mice using a modified two-step collagenase perfusion system kept at 37°C [15]. Briefly, mice were anesthetized with isoflurane and the portal vein was cannulated. Upon successful cannulation, the inferior vena cava (IVC) was immediately cut to allow fluid to drain. The liver was perfused at a flow rate of 8–10 ml/min for 5 min with calcium- and magnesium-free Hanks balanced salt solution. Digestion buffer containing low glucose DMEM (Sigma) supplemented with 100 U/ml type IV collagenase (Worthington), 15 mM HEPES, 1.8 mM CaCl_2_ and 100 U/ml penicillin-streptomycin was then perfused for an additional 10 min to digest the liver. Intermittent clamping of the IVC was performed to augment total cell yield. The liver was excised and cells were liberated by tearing and shaking of the liver with forceps followed by gentle trituration. The cell suspension was then filtered through a 70 µm strainer (BD Biosciences), washed by centrifugation thrice (50× g for 3 min at 4°C) and resuspended in isolation medium [high glucose DMEM (Sigma) supplemented with 15 mM HEPES (pH 7.4), 100 nM dexamethasone, 10% FBS and 100 U/ml penicillin-streptomycin. Cell viability was ≥90% as assessed by trypan blue exclusion. Cells were plated on collagen-coated (5 µg/cm^2^ Type 1 rat collagen; BD Biosciences) 6-well plates at 1 million cells per well. Cells were allowed to attach for 45 min at 37°C in a humidified 5% CO_2_ incubator before changing the medium to low glucose DMEM supplemented with 5 mM HEPES, 10 nM dexamethasone, 10% FBS and 100 U/ml penicillin-streptomycin. After 4 hours of attachment, the medium was replaced with serum-free low glucose DMEM supplemented with 5 mM HEPES, 10 nM dexamethasone, 20 ng/ml epidermal growth factor (EGF), 10 mM nicotinamide and 100 U/ml penicillin-streptomycin for overnight culture. Culture medium was changed daily. Medium was collected and stored at −20°C for analysis of urea production.

### Real-time PCR for arginase-1 expression

Tissues were pulverized in liquid nitrogen prior to RNA extraction. Total RNA was extracted with TRIzol reagent (Invitrogen), followed by RNA cleanup using an RNeasy Kit (Qiagen) as per the manufacturer's instructions. cDNA was synthesized from 1 µg of total RNA using an iScript cDNA synthesis kit (Bio-Rad). Gene expression was determined by quantitative PCR (Applied Biosystems Model 7500) using a TaqMan Gene Expression Assay specific for *Arg1* spanning exons 7 and 8 (Mm01190441_g1, Applied Biosystems) and *Arg2* (Mm00477592_m1, Applied Biosystems). Mouse 18s rRNA Taqman primer-probe set (Mm03928990_gl, Applied Biosystems) was used as internal reference for normalization. The reaction mixtures prepared in duplicate each contained 20× TaqMan mixture (Applied Biosystems), TaqMan primer-probe sets and 1 µl of cDNA template in a total volume of 20 µl. Reaction conditions: 2 min at 50°C and 10 min at 95°C, followed by 40 cycles at 95°C for 15 s and 60°C for 1 min. Relative gene expression was calculated using the comparative threshold method (2^−ΔΔCt^) and is presented as fold change of transcripts for *Arg1* or *Arg2* compared to 18s rRNA.

### Western blot analysis

Liver tissues were pulverized in liquid nitrogen and homogenized in ice-cold RIPA buffer (Millipore) including protease inhibitor cocktail (Roche). Twenty-micrograms of centrifuged, clarified protein samples were subjected to Western blot analysis and probed with rabbit polyclonal anti-Arg1 antibody (C-terminal) (1∶10000; Abcam, cat# ab91279) and mouse polyclonal anti-α-tubulin antibody (1∶5000, Sigma-Aldrich) used as loading control. Immunoreactive proteins were detected using horseradish peroxidase-conjugated goat anti-rabbit or anti-mouse secondary antibody (1∶5000, Sigma-Aldrich) and visualized by enhanced chemiluminescence detection (GE Healthcare).

### Urea production assay

Urea produced by hepatocytes was measured colorimetrically using a urea assay kit (Abnova) according to the manufacturer's instructions. Fresh culture media and known concentrations of urea were used as negative and positive controls, respectively. Urea reactions were performed in 96-well plates and concentrations were assessed using a plate reader (FLUOstar OPTIMA, BMG Labtech) at absorbance 520 nm.

### Biochemical analysis of plasma

Whole blood samples were obtained from the saphenous or submandibular vein prior to commencing the studies, as well as on day 4, 7 and 11 following the final tamoxifen (or vehicle) injection. Endpoint samples (on day 14 when the mice were sacrificed) were obtained by post-mortem cardiac puncture. Briefly, plasma collected in heparinized microhematocrit capillary tubes was separated from red blood cells by centrifugation and assayed immediately for ammonia concentration employing a commercial kit (Sigma-Aldrich) using 10–20 µl of plasma for each sample tested according to the manufacturer's instructions. The absorbance was measured with a spectrophotometer (Varian Cary 50 Bio UV-visible spectrophotometer) at 340 nm. The ammonia concentrations were calculated according to the non-competitive reaction equation.

For the guanidino compound analysis, mouse plasma was analyzed by HPLC cation-exchange chromatography using a Beckman Coulter HPLC system (Fullerton, California, USA) with post-column derivatization modified after Marescau *et al.* [16]. Plasma amino acids were analyzed by MassTrak Amino acid Analysis Solution on an Acquity UPLC System (Waters, Milford MA, USA). In brief, 5 µl of plasma sample was added to 10 µl of 5% albumin in saline solution to create a 3× dilution. Plasma proteins were precipitated by adding 15 µl of 10% sulfosalicylic acid solution containing the internal standard norvaline to the 15 µl of diluted plasma. Twenty microliters of derivatizing solution was added to the mixture and then heated at 55°C for 10 minutes prior to analysis.

### Arginase activity assay

Arginase activities of all samples were assayed as described previously [17]. Briefly, liver tissues were homogenized in a solution containing 40 µl of 0.1% Triton X-100 and 1× HALT protease inhibitor cocktail per mg of tissue. Lysate (10 µl) was diluted with water to a final volume of 100 µl, followed by the addition of 100 µl of 25 mM Tris-HCl (pH 7.5) and 20 µl of 10 mM MnCl_2_. Arginase was activated by heating each sample at 56°C for 10 min. Samples were then added with 100 µl of 0.5 M L-arginine (pH 7.9) as the substrate and incubated at 37°C for 1 h. Arginase enzyme activity will cause L-arginine to convert to urea. The conversion of arginine to urea was stopped by the addition of 900 µl of acid mixture [1∶3∶7 mixture of H_2_SO_4_ (96%)∶H_3_PO_4_ (85%)∶water]. Forty microliters of 9% solution of 1-phenyl-1,2-propanedione-2-oxime (ISPF) dissolved in ethanol was then added to each sample. The mixture was heated to 95°C for 30 min for development of a purple color for urea. Each sample was diluted 1∶6 with water and absorbance measured at 540 nm (Varian Cary 50 Bio UV-visible spectrophotometer). Urea standards ranging from 0.03 to 0.5 µmol were used to create a standard curve for each assay. The extent of arginase enzyme activity was normalized to the protein content of each lysate, as measured by a Bradford reagent assay using standard protocols.

### Statistical analysis

Survival curves were computed in each group of mice using the Kaplan-Meier method and compared across groups using the log rank test. All results are expressed as mean ± standard error of mean (SEM). Statistical analysis was performed using GraphPad Prism 6 (GraphPad Software, San Diego, California, USA). Means were compared using the two-tailed Student's t-*t*est. *P* values of <0.05 were considered statistically significant.

## Results

### Generation of an inducible Arg1 deficient mouse model

**Figure 1:**
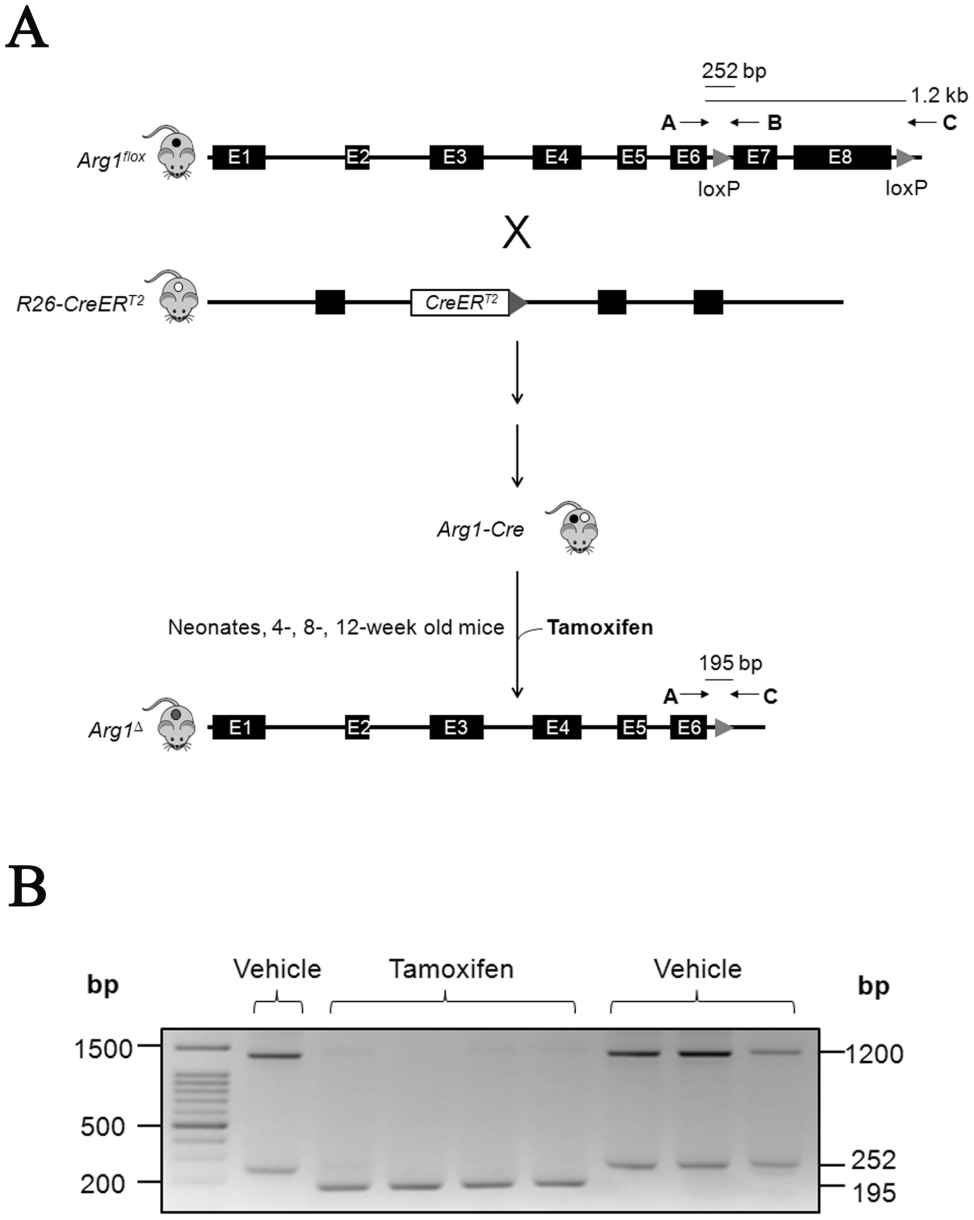
Generation of inducible Arg1-deficient mice. (A) Experimental setup for the gene targeting strategy. *Arg1^flox^* mice were crossbred with CreER^T2^ mice to generate Arg1-Cre mice. Four separate groups of mice have been tested for tamoxifen-mediated Cre removal of exons 7 and 8 of *Arg1*. The arrows depict the locations of primers used for genotyping the resulting mice with approximate sizes of the different PCR products shown. (B) Representative agarose gel of PCR genotyping using genomic DNA from ear punch or tail biopsies of vehicle- and tamoxifen-treated Arg-Cre mice. Arg1-Cre mice exhibited two bands at 1.2 kb and 252 bp, while knockout mice showed a single band at 195 bp.

Using a conditional targeting strategy, we generated an inducible Arg1-deficient mouse strain by crossing *Arg1^flox^* mice [12] with mice expressing a ligand-dependent chimeric Cre recombinase (CreER^T2^) (14). The “floxed” exons 7 and 8 of *Arg1* were then deleted through Cre-mediated loxP recombination with tamoxifen [13] to achieve nearly 100% penetrance of recombination. Three primers were used for genotyping four days after the last tamoxifen/vehicle administration (for details see ‘*Materials and Methods*’) (Fig. 1A) in 4 separate age groups of mice. As expected, *Arg1^flox^* mice produced 1.2 kb and 252 bp bands (indicative of intact exons 7 and 8). Successful excision of exons 7 and 8 of *Arg1* yields a band of 195 bp characteristic of the *Arg1* ^Δ^ allele (Fig. 1B).

### Verification of tamoxifen-induced inactivation of the *Arg1* gene

**Figure 2:**
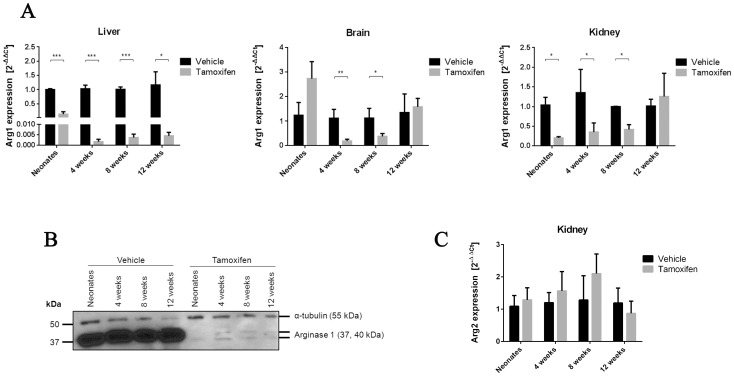
Down-regulation of Arg 1 expression in tamoxifen-treated Arg-Cre mice. (A) Transcriptional response of *Arg1* to tamoxifen induction. Real-time qPCR analysis of *Arg 1* gene expression was performed in liver, brain and kidney tissues 12 days after tamoxifen administration. Differences in RNA input for reverse transcription were normalized using Ct values obtained in parallel for mouse 18S rRNA. Fold change was calculated relative to the vehicle-treated samples using the comparative threshold method (2^−ΔΔCt^). Values are mean ± SEM for n = 3–6 in each group. Statistical significance between groups was determined by Student's *t*-test. *P<0.05, **P<0.01 and ***P<0.001. (B) Western blot analysis of total liver extracts (20 µg/well) from four age groups. Arg1 protein expression was evaluated by immunoblotting with an anti-Arg1 antibody (C-terminal) to confirm the level of knockdown. α-tubulin was used as loading control. Representative immunoblot from three independent experiments is shown. (C) Real-time qPCR analysis of *Arg 2* gene expression in kidney.

Real-time qPCR analysis was carried out to determine whether the deletion of exons 7 and 8 altered the transcriptional processing of *Arg1*. Indeed, data show markedly reduced *Arg1* transcripts, particularly in liver tissues of neonatal, 4-, 8- and 12-week tamoxifen-treated Arg-Cre mice (Fig. 2A). In other tissues (brain, kidney) where endogenous *Arg1* expression in wildtype mice is much lower than in liver, *Arg1* mRNA also decreased but variably in neonatal, 4- and 8-week tamoxifen-treated Arg-Cre mice. However, there was no significant change in Arg1 expression in brain and kidney in the 12-week group. In parallel, Western blots were performed to assess the expression of Arg1 protein, specifically in liver. As expected, immunoblotting results were in accordance with real-time PCR data. While vehicle-treated samples showed strong immunoreactive Arg1 bands, diminished expression of hepatic Arg1 was observed in tamoxifen-treated mice of all four age groups (Fig. 2B). Clearly, both qPCR and Western blot analysis verified that Cre-mediated recombination occurred after tamoxifen administration. In addition, measurement of renal Arg2 mRNA levels was carried out to examine if its expression was compensatorily modified in response to *Arg1* ablation. Unlike human ARG1-deficient patients with significant ARG2 induction [4], our results showed no statistically significant changes in renal Arg2 expression between the vehicle- and tamoxifen-treated Arg-Cre mice (Fig. 2C).

### Phenotypic characterization of inducible Arg1 deficient mice

**Figure 3:**
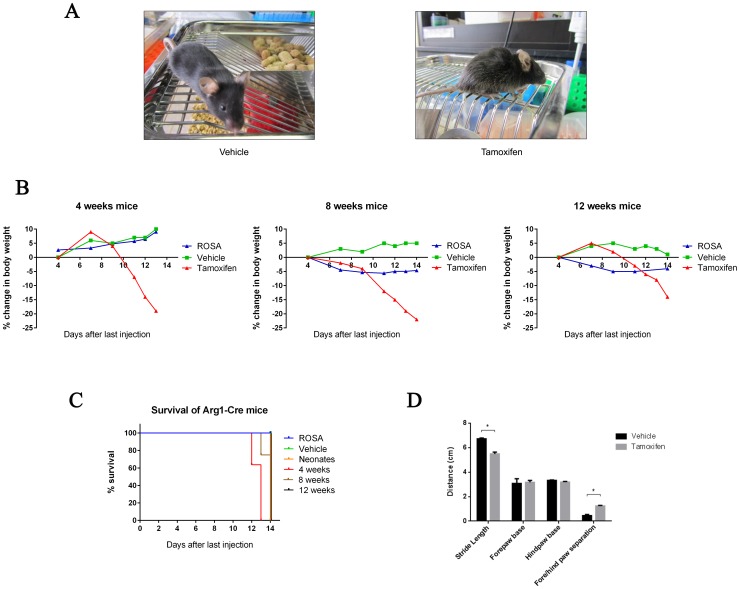
Impact of inducible Arg1 knockout on phenotypic presentation. (A) Typical appearance of Arg1 knockout mouse at humane endpoint (right), with healthy-appearing vehicle-treated control at the same timepoint (left). (B) Percent changes in body weight during the experimental period relative to body weights taken four days following injections are shown on y-axis. (C) Kaplan-Meier survival curve comparison depicts that tamoxifen-treated mice display significantly reduced survival rates when compared to ROSA and vehicle-treated mice. Data are mean ± SEM for n = 5–11 in each group. Statistical significance between groups was determined by Student's *t*-test (*P<0.05). (D) Quantitative analysis of walking footprint patterns based on measurements of stride length, forepaw base and hindpaw base width, and distance between front and hind footprint placement. (n = 6).

We next assessed the outcome of excision of *Arg1* exons 7 and 8 on phenotypic presentation and survival. Vehicle- and tamoxifen-treated Arg-Cre mice were grossly indistinguishable in phenotype from each other until approximately day 10 following the final tamoxifen injection. In tamoxifen-treated Arg-Cre mice, onset of phenotypic disparities were typically heralded by ruffled coat, dull eyes, and hunched body posture (Fig. 3A), followed by inactivity, progressive signs of ataxia and respiratory distress over the next 3–4 days. There were significant weight loss differences between the groups during the study period, although all mice were provided a standard chow diet *ad libitum*. In contrast to vehicle-treated mice, which generally showed age-appropriate weight gain, rapid weight loss was recorded in tamoxifen-treated mice starting at 7–10 days after the final tamoxifen injection. Growth disparity was most obvious in 4- and 8-week old mice, which exhibited up to 20% loss of body weight relative to day 4 from the last tamoxifen/vehicle injections, whereas ROSA mice exhibited little (in 8- and 12-week old mice) or no weight loss (in 4-week old mice) (Fig. 3B). In the last few days of life, when weight loss was progressing in tamoxifen-treated 8-week old mice, chow feeding went from baseline (5.4±0.5 g/day) to reduced (3.3±0.6 g/day) intake (n = 3), while in vehicle-treated mice feeding stayed constant (5.6±0.9 baseline vs 5.6±0.6 day 14 after last tamoxifen dose; n = 3).

Kaplan-Meier survival curves of tamoxifen-treated mice vs ROSA and vehicle-treated mice began to diverge on day 12 after the last injection and remained apart until the end of the study (Fig. 3C). In fact, the induced Arg1 deficient mice die about 2 weeks after tamoxifen administration, regardless of the starting age for excision of exons. In addition, gait abnormalities were also assessed quantitatively by analyzing the walking footprint patterns of the mice (Fig. 3D). Vehicle-treated mice walked with a steady alternating gait, whereas tamoxifen-treated mice adopted staggering movements showing irregularly spaced footprints. Although no changes were observed in the forepaw base and hindpaw base widths between the two groups, the tamoxifen-treated mice displayed a significantly shorter stride length and a greater distance between forepaw and hindpaw overlap, as compared with vehicle-treated mice.

### Effect of Arg-1 deficiency on liver functions

**Figure 4:**
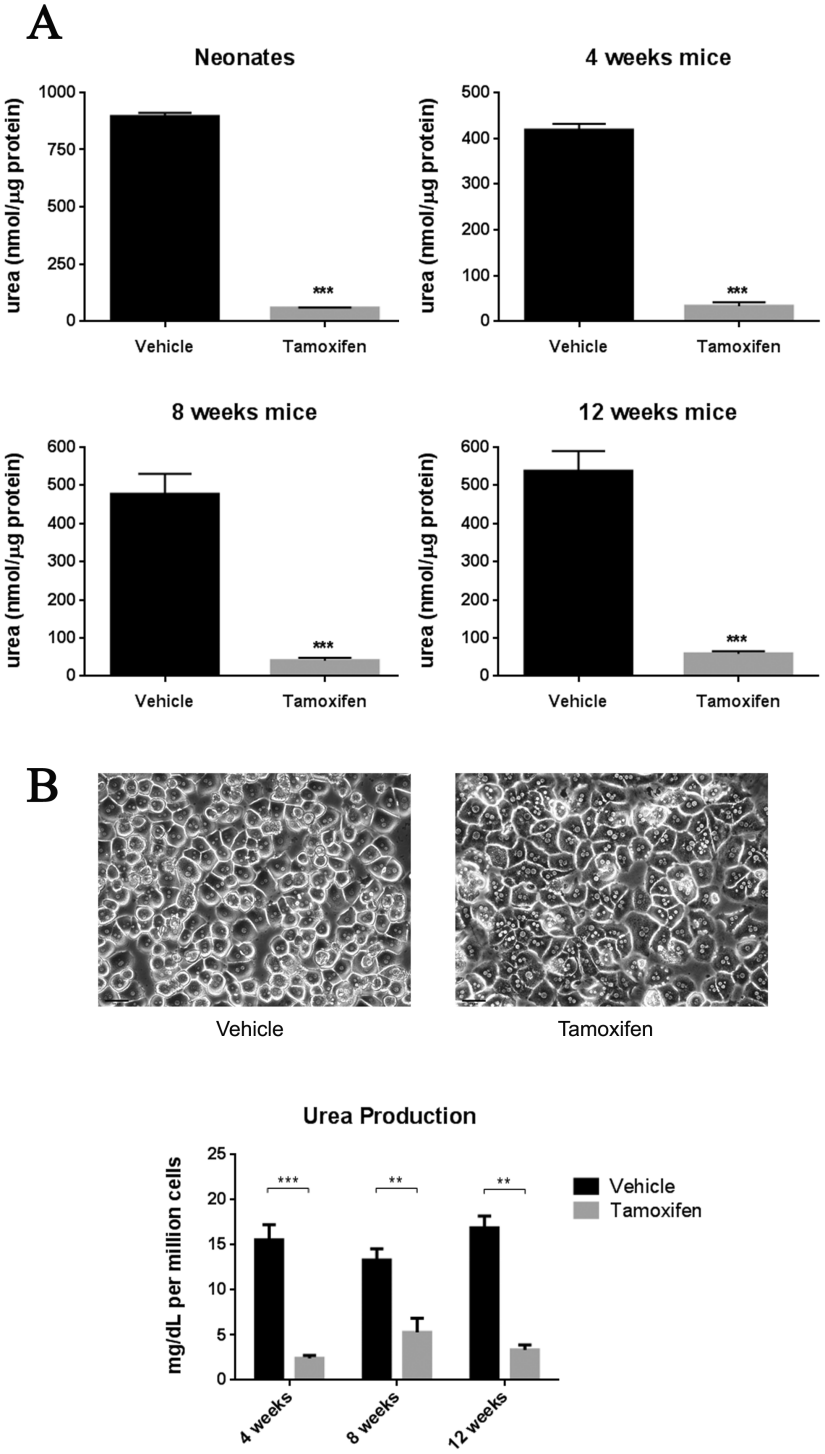
Reduced arginase enzyme activity and urea production in tamoxifen-treated Arg-Cre mice. (A) Arginase activity in liver tissue extracts from vehicle- and tamoxifen-treated mice. The livers were homogenized and the arginase enzyme activity assay was carried out as mentioned in the “*Materials and Methods*” section. (B) Functional capacity of hepatocytes assessed by determining urea production. Isolation of primary mouse hepatocytes was performed based on the two-step collagenase perfusion technique. Typical morphology of mouse hepatocytes cultured on a single layer of collagen gel at 24 h (top panel). Hepatocyte urea production was spectrophotometrically determined at 544 nm (bottom panel). Values are mean ± SEM for n = 3–7 in each group. *P<0.05, **P<0.01 and ***P<0.001.

To biochemically characterize the inducible *Arg1*-deficient mouse model, we performed arginase enzyme activity assays by quantifying the amount of urea produced from liver lysates of neonatal and adult mice aged between 4 to 12 weeks, treated with either vehicle or tamoxifen. Arg1 enzyme activity was significantly lower in the tamoxifen- treated group compared to the vehicle-treated mice at the neonatal stage (58.0±3.1 vs 895±16.6 nmoles of urea/µg protein), 4 weeks of age (33.4±8.2 vs 418±13.3 nmoles of urea/µg protein), 8 weeks of age (40.4±7.9 vs 476±53.9 nmoles of urea/µg protein) and 12 weeks of age (58.7±6.9 vs 537±52.9 nmoles of urea/µg protein) (Fig. 4A). To complement these studies, hepatic function was also assessed by examining the ability of hepatocytes *in vitro* to synthesize urea. Similar trends were observed in hepatocytes isolated from 4-, 8- and 12-week tamoxifen-treated mice, showing a nearly 3- to 5- fold reduction in urea production (Fig. 4B).

### Biochemical abnormalities in plasma of tamoxifen-treated Arg-Cre mice

**Figure 5:**
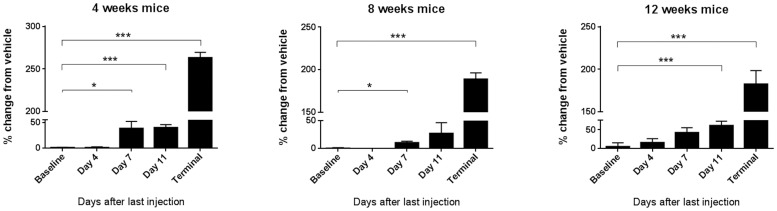
Tamoxifen-mediated inducible Arg1 knockout leads to hyperammonemic crisis. Plasma ammonia assay performed at five different time-points of three age groups. Values are expressed as percentage change in plasma ammonia concentration in tamoxifen-treated mice compared to vehicle-treated control mice. Ammonia concentrations ranged between 305–595 µmol/L (vehicle-treated mice) and 903–1791 µmol/L (tamoxifen-treated mice). Data are mean ± SEM for n = 3–5 in each group. *P<0.05, **P<0.01 and ***P<0.001.

Given that significant alterations in liver arginase activity and urea synthesis were observed, further biochemical studies were carried out. Plasma samples were collected from the three older age groups of vehicle- or tamoxifen-treated Arg-Cre mice and assayed for plasma ammonia concentration (PAC) at five different time-points: baseline (before treatment), and at days 4, 7, 11 and 13–14 (humane endpoint) after the last vehicle or tamoxifen injection. The percentage change in PAC in tamoxifen-treated vs vehicle-treated mice was then analyzed for all time-points across the three age groups (Fig. 5). The PAC for both the vehicle- and tamoxifen-treated groups was similar at baseline. Also, no major differences were evident at day 4 after the last injection. However, at days 7, 11 and endpoint after the last injection, the percentage change in PAC in tamoxifen-treated mice was significantly higher compared to that at baseline, in 4-week old (38.5±13.8; 40.1±6.0; 263±6.4%, respectively), 8-week old (10.5±2.6; 27.3±19.1; 189±7.5%) and 12-week old mice (42.4±13.2; 60.9±11.7; 182±15.9%, respectively).

Additionally, plasma amino acids and arginine metabolites of 4-, 8- and 12-week old mice were measured at baseline and humane endpoint. Compared to the vehicle-treated control mice, the levels in plasma of several amino acids were significantly perturbed in the tamoxifen-treated Arg-Cre mice (Table 1). Notably, the Arg1-induced knockout mice exhibited hyperargininemia with arginine levels nearly 8-fold greater than in “floxed” Arg1-Cre mice, which incidentally were 1.6-fold higher than in Cre mice (data not shown), presumably due to the loxP sites within the introns partially affecting Arg1 expression. The induced Arg1 knockout mice also showed an approximately 1.5–2.3-fold increase in citrulline and guanidinoacetic acid (GAA), whereas proline was reduced about 50% compared to the control vehicle-treated mice. Whilst no significant differences were observed in glutamine, aromatic amino acids and acidic amino acids, alanine, asparagine, glycine, methionine, serine, threonine, isoleucine, leucine and valine were all reduced (about 25–50%) in the induced Arg1 knockout mice. The plasma level of ornithine, the product of Arg1 activity along with urea, however, was not significantly affected.

**Table 1:** Plasma amino acid analysis of mice from different age groups (pooled) measured at humane endpoint.

		Vehicle-treated mice	Tamoxifen-treated mice	*P* value
BCAA	Valine	204±17.5	124±8.0	<0.001
	Isoleucine	71.7±4.0	51.9±4.1	<0.01
	Leucine	146±13.4	98.0±7.3	<0.01
Aromatic	Phenylalanine	108±13.2	90.7±4.2	NS
	Tryptophan	94.6±11.3	83.7±7.9	NS
	Tyrosine	79.2±11.3	55.3±3.7	NS
Heterocyclic	Proline	99.1±7.3	52.6±2.0	<0.001
Carboxamide	Asparagine	56.1±9.4	30.3±1.2	<0.05
	Glutamine	700±38.8	684±29.7	NS
Hydroxy	Serine	147±13.9	81.8±3.5	<0.001
	Threonine	188±13.1	89.0±3.6	<0.001
Monoamino, monocarboxylic	Alanine	451±37.3	211±19.0	<0.001
	Glycine	244±18.7	138±6.7	<0.001
Diamino, monocarboxylic	Histidine	97.6±14.3	72.5±4.4	NS
	Lysine	318±31.1	342±41.5	NS
Monoamino, dicarboxylic	Aspartic acid	19.6±1.6	17.9±2.0	NS
	Glutamic acid	54.9±6.0	44.4±5.4	NS
Urea cycle-related	Arginine	100±7.9	784±62.1	<0.001
	Citrulline	61.4±2.7	89.7±4.5	<0.001
	Ornithine	78.7±7.5	70.0±20.5	NS
Thioether	Methionine	81.2±8.1	43.0±3.7	<0.001
Guanidino compound	Guanidino acetic acid	1.2±0.1	2.8±0.3	<0.001

Values are mean ± SEM for n = 11–12 and are expressed as micromoles per liter (µmol/L). BCAA, branched chain amino acid; NS, not significant.

## Discussion

Our understanding of the pathophysiologic processes underlying Arg-1 deficiency can be greatly enhanced by the development of appropriate knockout animal models. The first Arg1 knockout mouse model was generated in 2002 by Iyer and coworkers by replacing the active site of the *Arg1* gene (exon 4) with a neomycin resistance cassette [11]. However, the mice died before weaning and were not amenable to further studies on somatic growth and neurological development. To overcome these limitations, we developed a novel time-dependent control of *in vivo Arg1* gene disruption using a tamoxifen-inducible Cre/loxP conditional knockout strategy. Arg1 knockout mice spanning the neonatal period to early adulthood were generated and demonstrated significant deficits of Arg1 mRNA and protein expression in various tissues with the effect most pronounced in liver where Arg1 expression was reduced to barely detectable levels. The unchanged level of Arg1 mRNA level in the brain and kidney tissues of the 12-week old tamoxifen-treated mice may be due to variable efficacy of tamoxifen to reach estrogen receptor targets in these tissues in older mice to induce Cre-mediated excision or to altered chromatin structure at the *Arg1* locus in these tissues that affects excision efficiency.

Tamoxifen-treated Arg1-deficient mice exhibited progressive weight loss and a neurological phenotype consistent with overt loss of motor function with some aspects similar to human ARG1 deficiency [18]. Since the neuromotor deficits were observed before severe weight loss, the lack of uniformity of step alternation in Arg1-deficient mice is likely a consequence of *Arg1* gene disruption rather than changes in body weight. Nevertheless, more specific tests at different time points would allow further appraisal of this presumption. However, in contrast to human patients who can often survive longterm, these mice died invariably by two weeks after the last tamoxifen injection. Tamoxifen has been associated with several side effects including weight loss due to its direct toxic effect on gastric mucosa, thereby interfering with the normal digestive processes [19]. Such effects are likely to cause under nutrition with a consequent deleterious effect on body weight and growth. To examine the effect of tamoxifen, ROSA mice (that express the Cre transgene but do not possess floxed *Arg1* alleles) treated with tamoxifen were also included in our early experiments. These mice showed only a minor decrement in weight loss (about 5% in 8- and 12-week old mice and no change in 4-week old mice). Most importantly, this minimal degree of weight loss did not affect survival rate, hence, excluding major toxicity of tamoxifen in our studies.

Symptoms developed in ARG1-deficiency human patients are generally less severe compared to other urea cycle disorders. The overexpression of ARG2 is a potential compensatory response, which can attenuate disease progression [1], [4]. However, we found no statistically significant evidence for renal Arg2 compensation in our inducible Arg1 knockout mice resulting in a severe manifestation of disease in mice compared to humans. Nevertheless, the mice generated in our studies have proven highly useful for proof-of-concept studies by exhibiting several hallmark presentations of Arg-1 deficiency such as impaired hepatic arginase activity and hyperargininemia.

Various degrees of metabolic derangements were caused by the inducible knockout of Arg1 activity. The level of free ammonia in plasma was significantly increased in Arg1-deficient mice, but glutamine which functions as temporary ‘repository’ for ammonia was not significantly affected. The accumulation of ammonia is a deleterious consequence of impaired ureagenesis and leads to toxicity in the central nervous system (CNS). Interestingly, other amino acids such as alanine, asparagine, glycine, methionine, proline, serine and threonine, which play roles in the incorporation of ammonia nitrogen were all significantly reduced. These observations are paradoxical, suggesting an alternative ammonia-scavenging pathway may be involved. In addition to hyperammonemia, profound hyperargininemia was present in tamoxifen-treated mice accompanied by modestly elevated guanidinoacetic acid (GAA) and the liver urea cycle intermediate citrulline. The guanidino group of arginine is required to yield guanidinoacetic acid (GAA), and subsequently creatine and creatinine via the transaminidation pathway. Clearly, ammonia is not solely responsible for the neurologic insult in these mice. In fact, elevated arginine and guanidino compounds may also result in neurotoxicity via augmented nitric oxide synthesis, thereby leading to oxidative damage to the brain [20]. Indeed, several guanidino compounds were previously shown to be increased in plasma, cerebrospinal fluid (CSF), as well as brain tissue of hyperargininemic patients [21], [22] and in the mouse model [23].

Another finding of this study is a significant 50% reduction of plasma proline concentration in Arg1-deficient mice. Proline has the potential to suppress reactive oxygen species (ROS) and could contribute to aberrant redox homeostasis [24]. Due to its neuroprotective role, plasma proline is a promising biomarker of neurological deficits [25]. Taken together, the deficit in coordinated neuromotor abilities observed in inducible Arg-1 deficient mice is likely resulting from a combinatorial effect of hyperammonemia, accumulation of arginine and its metabolites, such as guanidino compounds, as well as alterations of other amino acids. Additional studies will be required to elucidate the mechanisms of cognitive impairment in the brain. Elevated arginine and ammonia concentrations have been reported to be associated with reduced concentrations of branched-chain amino acids (BCAA) such as isoleucine, leucine and valine [26], [27], which is in line with our observations. Hyperargininemia and hyperammonemia promote BCAA degradation and catabolism, respectively, for lean tissue growth by enhancing mitochondrial oxidative function in skeletal muscle. Further investigations examining whole-body composition, as well as energy expenditure in the control and inducible Arg1-deficient mice could reveal clues to the mechanisms for the observed phenotypes.

Notably, the abnormalities in most amino acid levels seen in our tamoxifen-treated mice coincided with the growth retardation phase that they incurred about 10 days following tamoxifen administration. These mice demonstrated failure to thrive, followed by rapid weight loss and early mortality. There are a few possibilities for this observation. First, an adaptive homeostatic mechanism may be initiated in response to changes in amino acid concentration in the body. When the capacity to degrade excess amino acids is impaired, a mechanism for modification of feeding behavior will come into play. According to the aminostatic hypothesis, an imbalance of plasma amino acids may trigger satiety signals in the brain, thereby affecting food consumption [28]. It was later demonstrated that increased plasma amino acids results in a waning of appetite, suggesting an implication of an appetite-controlling mechanism [29], which is initiated by the hypothalamic neuronal circuitry [30]. In fact, there is a reciprocal causal relationship between plasma amino acid concentrations and appetite, where hunger is suppressed as amino acid levels rises. In this context, there is a possibility that the tremendous rise of arginine in our Arg1-deficient mice has initiated a signal to reduce food intake that leads to subsequent body weight loss. Prolonged low level of food intake likely affects amino acid homeostasis, as reflected in the altered plasma amino acids, which may eventually be fatal for the induced knockout mice. A second explanation involves an anorexigenic effect (appetite-suppressing) due to marked elevation in plasma ammonia. Ammonium ion serves as a regulator of insulin secretion where the latter is a potent anorexigenic hormone [31]. The Arg1 deficiency-elicited rise in plasma ammonia could indirectly reshape feeding patterns through the effect of insulin. More importantly, ammonia is mainly produced in the gut before transport to the liver for detoxification. At this time, we are uncertain whether body weight loss is the main precipitating cause of death in the tamoxifen-treated Arg1 knockout mice or if it is due to ammonia elevation, or a combination of both factors.

Conceivably, the plasma ornithine concentration should also decrease as a result of reduced flux through Arg1. The results from the present study however, show that ornithine was not significantly altered in our knockout mice, arguing against Arg1 in the regulation of ornithine production. This was an unexpected finding and is in contrast to the original Arg1 knockout model, which exhibited hypoornithinemia [11]. There is a potential explanation in that the ornithine aminotransferase reaction converts ornithine to L-1-pyrroline-5-carboxylate (5PC), an intermediate of proline synthesis. In humans, this reaction is almost irreversible, except in the neonatal period, towards 5PC formation, but in mice it could be possible that there is a reverse flux that leads to ornithine formation by consuming proline. This would explain both the low proline and normal ornithine levels. Many of the symptoms found in human ARG1-deficiency can be partially alleviated through ornithine supplementation. However, our attempts to ameliorate the biochemical consequences of Arg1 deficiency with ornithine in the drinking water were unsuccessful, despite markedly increased ornithine levels (data not shown). From these observations, it is postulated that arginase inactivity may be compensated by other unknown mechanisms regulating plasma ornithine levels.

After we had written this manuscript, Kasten et al. [32] reported a similar mouse model using a different tamoxifen regimen (single oral dose of about 4 mg vs five × 1 mg daily i.p. doses in 4-, 8-, 12- week old mice or two intragastric administrations in neonatal mice in our studies), which resulted in inconsistent recombination and variable loss of Arg1 activity in [32] vs virtually complete knockout of Arg1 in our studies in all mice. Humane endpoints differed substantially (>30% loss of body weight [32] vs >15% here). Therefore, survival was gauged as longer in [32] at 21.5 days vs 19 days (as counted from the first day of tamoxifen administration or 14 days from the last day) in our studies. There were some differences in plasma amino acids and ornithine levels that may be partially associated with the different blood sampling methods used [33]. In contrast to the retro-orbital plexus bleeding technique employed in [32], we collected blood via the submandibular facial vein, which required only momentary restraint without the need for anesthesia, thereby eliminating potential drug effects on metabolic parameters and allowed for repeated blood sampling at multiple time points particularly to monitor the changes in plasma ammonia.

In conclusion, we present here a novel animal model for studying Arg1-deficiency at any time point during the normal lifespan of a mouse. The inducible Arg1 knockout mice displayed a unique biochemical phenotype. Titration of the extent of *Arg1* gene disruption by modulating the typical tamoxifen administration regimen [13] may yield mice that survive much longer and display typical neurological characteristics observed in human ARG1 deficiency. Additionally, gene therapeutic approaches similar or distinct from those tested recently [17], [34], [35] can be attempted and will allow for the study of the exciting roles for Arg1 in immune function, as well as in the pathophysiology of cardiovascular disease, asthma and cancer.

## References

[1] Iyer RK, Jenkinson CP, Vockley JG, Kern RM, Grody WW, et al (1998) The human arginases and arginase deficiency. J Inherit Metab Dis 21: 86–100. 10.1023/a:1005313809037 9686347

[2] Morris SM Jr, Bhamidipati D, Kepka-Lenhart D (1997) Human type II arginase: sequence analysis and tissue-specific expression. Gene 193: 157–161. 9256072 10.1016/s0378-1119(97)00099-1

[3] Vockley JG, Jenkinson CP, Shukla H, Kern RM, Grody WW, et al (1996) Cloning and characterization of the human type II arginase gene. Genomics 38: 118–123. 8954792 10.1006/geno.1996.0606

[4] Grody WW, Kern RM, Klein D, Dodson AE, Wissman PB, et al (1993) Arginase deficiency manifesting delayed clinical sequelae and induction of a kidney arginase isozyme. Hum Genet 91: 1–5. 8454280 10.1007/BF00230212

[5] Crombez EA, Cederbaum SD (2005) Hyperargininemia due to liver arginase deficiency. Mol Genet Metab 84: 243–251. 15694174 10.1016/j.ymgme.2004.11.004

[6] Batshaw ML, MacArthur RB, Tuchman M (2001) Alternative pathway therapy for urea cycle disorders: twenty years later. J Pediatr 138: S46–S55. 11148549 10.1067/mpd.2001.111836

[7] Sparkes RS, Dizikes GJ, Klisak I, Grody WW, Mohandas T, et al (1986) The gene for human liver arginase (ARG1) is assigned to chromosome band 6q23. Am J Human Genet 39: 186–193. 3752085 PMC1683935

[8] Takiguchi M, Haraguchi Y, Mori M (1988) Human liver-type arginase gene: structure of the gene and analysis of the promoter region. Nucleic Acids Res 16: 8789–8802. 3174433 10.1093/nar/16.18.8789 PMC338635

[9] Dizikes GJ, Spector EB, Cederbaum SD (1986) Cloning of rat liver arginase cDNA and elucidation of regulation of arginase gene expression in H4 rat hepatoma cells. Somat Cell Mol Genet 12: 375–384. 3461568 10.1007/BF01570732

[10] Haraguchi Y, Takiguchi M, Amaya Y, Kawamoto S, Matsuda I, et al (1987) Molecular cloning and nucleotide sequence of cDNA for human liver arginase. Proc Natl Acad Sci 84: 412–415. 3540966 10.1073/pnas.84.2.412 PMC304217

[11] Iyer RK, Yoo PK, Kern RM, Rozengurt N, Tsoa R, et al (2002) Mouse model for human arginase deficiency. Mol Cell Biol 22: 4491–4498. 12052859 10.1128/MCB.22.13.4491-4498.2002 PMC133904

[12] El Kasmi KC, Qualls JE, Pesce JT, Smith AM, Thompson RW, et al (2008) Toll-like receptor-induced arginase 1 in macrophages thwarts effective immunity against intracellular pathogens. Nat Immunol 9: 1399–1406. 18978793 10.1038/ni.1671 PMC2584974

[13] Feil S, Valtcheva N, Feil R (2009) Inducible cre mice. Methods Mol Biol 530: 1–21. 19266339 10.1007/978-1-59745-471-1_18

[14] Soriano P (1999) Generalized lacZ expression with the ROSA26 Cre reporter strain. Nat Genet 21: 70–71. 9916792 10.1038/5007

[15] Klaunig JE, Goldblatt PJ, Hinton DE, Lipsky MM, Chacko J, et al (1981) Mouse liver cell culture. I. Hepatocyte isolation. In Vitro 17: 913–925. 6273298 10.1007/BF02618288

[16] Marescau B, Deshmukh DR, Kockx M, Possemiers I, Qureshi IA, et al (1992) Guanidino compounds in serum, urine, liver, kidney, and brain of man and some ureotelic animals. *Metabolism* 41: 526–532. 1588833 10.1016/0026-0495(92)90213-t

[17] Gau CL, Rosenblatt RA, Cerullo V, Lay FD, Dow AC, et al (2009) Short-term correction of arginase deficiency in a neonatal murine model with a helper-dependent adenoviral vector. Mol Ther 17: 1155–1163. 19367256 10.1038/mt.2009.65 PMC2835205

[18] Prasad AN, Breen JC, Ampola MG, Rosman NP (1997) Argininemia: a treatable genetic cause of progressive spastic diplegia simulating cerebral palsy: case reports and literature review. J Child Neurol 12: 301–309. 9378897 10.1177/088307389701200502

[19] Huh WJ, Khurana SS, Geahlen JH, Kohli K, Waller RA, et al (2012) Tamoxifen induces rapid, reversible atrophy, and metaplasia in mouse stomach. Gastroenterology 142: 21–24. 22001866 10.1053/j.gastro.2011.09.050 PMC3708546

[20] Luiking YC, Engelen MPKJ, Deutz NEP (2010) Regulation of nitric oxide production in health and disease. Curr Opin Clin Nutr Metab Care 13: 97–104. 19841582 10.1097/MCO.0b013e328332f99d PMC2953417

[21] Mizutani N, Hayakawa C, Ohya Y, Watanabe K, Watanabe Y, et al (1987) Guanidino compounds in hyperargininemia. Tohoku J Exp Med 153: 197–205. 3433275 10.1620/tjem.153.197

[22] Deignan JL, De Deyn PP, Cederbaum SD, Fuchshuber A, Roth B, et al (2010) Guanidino compound levels in blood, cerebrospinal fluid, and post-mortem brain material of patients with argininemia. Mol Genet Metab 100: S31–36. 20176499 10.1016/j.ymgme.2010.01.012

[23] Deignan JL, Marescau B, Livesay JC, Iyer RK, De Deyn PP, et al (2008) Increased plasma and tissue guanidino compounds in a mouse model of hyperargininemia. Mol Genet Metab 93: 172–178. 17997338 10.1016/j.ymgme.2007.09.016

[24] Krishnan N, Dickman MB, Becker DF (2008) Proline modulates the intracellular redox environment and protects mammalian cells against oxidative stress. Free Radic Biol Med 44: 671–681. 18036351 10.1016/j.freeradbiomed.2007.10.054 PMC2268104

[25] Louin G, Neveux N, Cynober L, Plotkine M, Marchand-Leroux C, et al (2007) Plasma concentrations of arginine and related amino acids following traumatic brain injury: proline as a promising biomarker of brain damage severity. Nitric Oxide 17: 91–97. 17613263 10.1016/j.niox.2007.05.006

[26] Jobgen W, Meininger CJ, Jobgen SC, Li P, Lee MJ, et al (2009) Dietary L-arginine supplementation reduces white fat gain and enhances skeletal muscle and brown fat masses in diet-induced obese rats. J Nutr 139: 230–237. 19106310 10.3945/jn.108.096362 PMC3151442

[27] Holecek M, Kandar R, Sispera L, Kovarik M (2011) Acute hyperammonemia activates branched-chain amino acid catabolism and decreases their extracellular concentrations: different sensitivity of red and white muscle. Amino Acids 40: 575–584. 20614225 10.1007/s00726-010-0679-z

[28] Mellinkoff SM, Frankland M, Boyle D, Greip M (1956) Relationship between serum amino acid concentration and fluctuations in appetite. J Appl Physiol 8: 535–538. 13295170 10.1152/jappl.1956.8.5.535

[29] Harper AE, Peters JC (1989) Protein intake, brain amino acid and serotonin concentrations and protein self-selection. J Nutr 5: 677–689. 10.1093/jn/119.5.677 2656935

[30] Berthoud HR (2002) Multiple neural systems controlling food intake and body weight. Neurosci Biobehav Rev 26: 393–428. 12204189 10.1016/s0149-7634(02)00014-3

[31] Feldman JM, Lebovitz HE (1971) Ammonium ion, a modulator of insulin secretion. Amer J Physiol 221: 1027–1032. 4329423 10.1152/ajplegacy.1971.221.4.1027

[32] Kasten J, Hu C, Bhargava R, Park H, Tai D, et al. (July 6, 2013)Lethal phenotype in conditional late-onset arginase 1 deficiency in the mouse. Mol Genet Metab DOI: 10.1016/j.ymgme.2013.06.020. 10.1016/j.ymgme.2013.06.020 PMC3800271 23920045

[33] Fernández I, Peña A, Del Teso N, Pérez V, Rodríguez-Cuesta J (2010) Clinical biochemistry parameters in C57BL/6J mice after blood collection from the submandibular vein and retroorbital plexus. J Am Assoc Lab Anim Sci 49: 202–206. 20353696 PMC2846009

[34] Lee EK, Hu C, Bhargava R, Rozengurt N, Stout D, et al (2012) Long-term survival of the juvenile lethal arginase-deficient mouse with AAV gene therapy. Mol Ther 20: 1844–1851. 22760543 10.1038/mt.2012.129 PMC3464644

[35] Lee EK, Hu C, Bhargava R, Ponnusamy R, Park H, et al (2013) AAV-based gene therapy prevents neuropathology and results in normal cognitive development in the hyperargininemic mouse. Gene Ther 20: 785–796. 23388701 10.1038/gt.2012.99 PMC3679314

